# Hyperthyroidism and the risk of non-thyroid cancer: a Danish register-based long-term follow-up study

**DOI:** 10.1530/ETJ-23-0181

**Published:** 2024-04-01

**Authors:** Thea Riis, Steen Joop Bonnema, Thomas Heiberg Brix, Lars Folkestad

**Affiliations:** 1 Department of Endocrinology, Odense University Hospital, Institute of Clinical Research, University of Southern Denmark, Odense, Denmark

**Keywords:** hyperthyroidism, Graves’ disease, toxic nodular goiter, cancer, epidemiology

## Abstract

**Objective:**

Cancer is the second most common cause of death worldwide. It is currently debated whether thyroid dysfunction is a modifiable cancer risk factor. Our aim was to evaluate the risk of cancer in patients with hyperthyroidism.

**Methods:**

This is a register-based nationwide cohort study of individuals with a diagnosis of hyperthyroidism. Each hyperthyroid case was matched with four reference individuals according to age and sex. Using Fine and Gray competing risk regression models, we studied the association of hyperthyroidism and subsequent all-cause cancer diagnoses, adjusted for preexisting morbidity. Sub-analyses were stratified for cause of hyperthyroidism (Graves’ disease and toxic nodular goiter, age when diagnosed with hyperthyroidism, sex, and cancer localization (lung, prostate, breast, and colorectal cancer)).

**Results:**

The cohort consisted of 95,469 patients with hyperthyroidism (followed for a median of 10.9 years (range: 5.2–17.2)), and 364,494 reference individuals (followed for a median of 11.2 years (range: 5.4–17.4)). Hyperthyroidism was associated with increased all-cause cancer risk (sub-distribution hazard ratio (SHR): 1.12; 95% CI: 1.10–1.14), as well as an increased risk of breast (SHR: 1.07; 95% CI: 1.02–1.13), lung (SHR: 1.20; 95% CI: 1.16–1.26), and prostate cancer (SHR: 1.10; 95% CI: 1.02–1.19), but not colorectal cancer (SHR: 1.04; 95% CI: 0.99–1.09). Sub-analyses stratified for age when diagnosed with hyperthyroidism and cause of hyperthyroidism yielded similar results.

**Conclusion:**

In this register-based study, patients with hyperthyroidism had an increased risk of cancer, in particular lung, prostate, and breast cancer. Whether a causal link exists remains to be proven.

## Introduction

Cancer is the second most common cause of death worldwide and imposes a burden both individually and to the health care systems (1). It has been suggested that four out of ten cancer cases are preventable (2). Therefore, identifying and reducing modifiable risk factors for cancer, such as smoking, obesity, or alcohol intake, is of great importance. Whether thyroid dysfunction is a modifiable risk factor is currently debated.

Several *in vitro* studies have demonstrated an effect of thyroid hormones on cancer cell growth (3, 4, 5, 6, 7). *In vivo* studies on mice and rats have shown that thyroid hormones induce proliferation and growth of cancer cells as well as promoting tumor angiogenesis (8, 9, 10, 11, 12). It is unsettled whether these pathophysiological observations can translate into an increased risk of non-thyroid cancer in patients with hyperthyroidism (13). Results from some (14, 15, 16, 17, 18), but not all (19, 20, 21), population-based studies support that an association exists between hyperthyroidism and cancer, especially lung (15, 16, 17), breast (15, 16, 18), and prostate cancer (14, 15). Other studies, investigating hyperthyroidism and the risk of hepatocellular (19) or gastrointestinal cancer (20), found no such association. In a study by Krashin *et al.* (17), hyperthyroidism was associated with a decreased risk of colorectal and prostate cancer, but an increased risk of lung cancer. Apart from that study (17), previous studies focused exclusively on middle-aged to elderly patients. In addition, common for these previous studies were limited statistical power due to relatively small sample sizes and/or insufficient length of follow-up, and the lack of competing risk analyses.

The personal identification number (CPR number) assigned to all inhabitants in Denmark allows for individual record linkage between different governmental registries (22). The Danish health care system is uniform, tax financed, and covers all residents. These conditions allow complete ascertainment and long-term follow-up of numerous clinical conditions (23), thereby limiting some of the shortcomings present in previous studies.

We hypothesized that patients with hyperthyroidism have an increased risk of being diagnosed with cancer compared to euthyroid individuals. We used the nationwide Danish health registries (22, 24) to evaluate the all-cause cancer risk in patients diagnosed with hyperthyroidism, as well as the risk of being diagnosed with one of four of the most frequent cancers, i.e. lung, prostate, breast, and colorectal cancer. Furthermore, we investigated whether the cancer risk is dependent on the cause of hyperthyroidism, i.e. Graves’ disease (GD) and toxic nodular goiter (TNG), the localization of cancer, sex, and age when hyperthyroidism was first diagnosed.

## Methods

### Data sources

The study cohort was created using the Danish Civil Registration System and The Danish National Patient Register (DNPR). The Danish Civil Registration System was established in 1968 and covers information on demographics and date of death of all people who are living or have lived in Denmark (22). The DNPR includes primary and supplementary diagnosis for all hospitalizations since 1977, and outpatient visits (including emergency room visits) since 1995 (24). Discharge diagnoses for all contacts, since 1995, are registered according to the International Classification of Diseases version 10 (ICD-10). The register has a coverage above 99%, and the overall positive predictive value of the diagnosis of cancer and hyperthyroidism is above 95% (23).

### Design and identification of the study population

The study is a register-based nationwide cohort study. Individuals ≥18 years were eligible for this study if registered in the DNPR with an ICD-10 code reflecting hyperthyroidism (ICD-10 codes E05.0-9, E06.2, E06.4, H05.2, and H06.2) between January 1, 1995, and December 31, 2018. The index date was defined as the date on which the first diagnosis of hyperthyroidism was entered into the DNPR. For each hyperthyroid individual, four reference individuals (matched for birth month, birth year, and sex) were randomly selected from the Danish general population via the Danish Civil Registration System (22). Reference individuals were included if they were alive at the index date and not diagnosed with goiter, hyper- or hypothyroidism (ICD-10 codes E04.0-9, E.07.0-9, E05.0-2, E05.9, H06.2, E03.0-9, E89.9, respectively) during the study period. The Supplementary Table 1 (see section on supplementary materials given at the end of this article) summarizes all relevant data sources and ICD-10 diagnoses used to identify patients with hyperthyroidism and patients with cancer.

We excluded all hyperthyroid patients with a cancer diagnosis entry in the DNPR prior to the index date. We also excluded all matched reference individuals for these excluded individuals. If a reference individual was diagnosed with cancer before the index date, this individual was excluded from the analysis. All included individuals were followed prospectively from the index date until migration, death, a registered diagnosis of cancer in the DNPR, or end of study, whichever came first.

### Study outcome measures

The primary outcome was risk of all-cause cancer following hyperthyroidism, while the secondary outcomes were risks of breast, prostate, lung, and colorectal cancer. All-cause cancer was defined as a diagnosis of any first cancer, including non-melanoma skin cancer but excluding thyroid cancer. A link between hyperthyroidism and thyroid cancer has been reported, but whether this is causal or due to increased surveillance in patients with hyperthyroidism is debated (25, 26). Since the register-based approach used in the present study does not allow for a sufficient handling of this kind of bias, the inclusion of thyroid cancer as an outcome could falsely exaggerate any association between hyperthyroidism and thyroid cancer. Definitions are detailed in the Supplementary Table 1. The date of cancer was defined as the date of the first entry in the DNPR with a cancer diagnosis. The burden of pre-existing morbidity was assessed using the Charlson Comorbidity Index (CCI) on the index date, as described by Christensen *et al.* (27). The CCI includes 19 disease categories, each assigned a score from 0 to 3 depending on severity. For each individual, the CCI score is the sum of the scores for all of the respective conditions.

### Statistical analysis

Data are presented as mean (± SD), median (interquartile range (IQR)), number of events, and percent of the population, as appropriate. To evaluate the sub-distribution hazard ratio (SHR), we fitted a Fine and Gray semiparametric competing risk regression model (28). The SHR was considered statistically significant if the 95% confidence interval (CI) did not include the value 1.00. The assumption of proportional hazards was evaluated by the log-log plot of survival for each group within each model used, accepting that the assumption of proportionality held, if the plotted lines did not cross.

The risks of all-cause cancer, lung cancer, breast cancer, colorectal cancer, and prostate cancer in all hyperthyroid patients, patients with TNG, or patients with GD compared to the general population were evaluated and presented as SHRs. All men were excluded in the risk analysis of breast cancer. Likewise, all women were excluded in the risk analysis of prostate cancer. All analyses were adjusted for age, sex, and the burden of comorbidity by CCI.

Common symptoms for both cancer and hyperthyroidism include fatigue and weight loss. Therefore, the risk of Berkson’s bias, i.e. the risk that a diagnosis of one disease (hyperthyroidism) leads to the increased risk of being diagnosed with another disease (cancer) during the work-up of the primary diagnosis, is not negligible in our study. We accounted for this bias by applying several censoring windows chosen arbitrarily (i.e. 3 months, 1 year, or 3 years between hyperthyroidism and the cancer diagnosis, respectively).

Further, the risk of bias due to excess morbidity and mortality in patients with hyperthyroidism is potentially high in studies evaluating the consequences of having or having had hyperthyroidism. As hyperthyroidism is associated with increased mortality (29), hyperthyroid individuals, at least theoretically, could have a shorter observation period than the reference individuals and consequently, a lower absolute risk of being diagnosed with cancer (30). We adjusted for this competing risk of death using the Fine and Gray competing risk regression model (28) and computed a cause-specific cumulative incidence function. This provides the absolute (or crude) risk of having the event (cancer) by time *t*, accounting for the fact that it is impossible to have the event if a competing event (death) occurs first.

Finally, differences in baseline risk of cancer may induce additional bias. Sex and age are major risk factors for both cancer and hyperthyroidism. We have, by design, taken this into account and matched our reference population by gender and birth month and year. In an attempt to adjust for pre-existing morbidity, risk estimate analyses were performed using the CCI. All statistical analyses were done using Stata 17 (StataCorp, College Station, TX, USA).

### Ethical considerations

The investigators were blinded to the identity of patients and the reference population. To ensure participant confidentiality, results are not shown if the number of participants in a subgroup was below five. The study was approved by Statistics Denmark and the Danish Health Data Authority (reference no. 704047).

## Results

We identified 101,043 patients with hyperthyroidism and 404,170 reference subjects. We excluded 5574 hyperthyroid individuals, as well as the corresponding 20,288 reference individuals, due to cancer being diagnosed prior to the hyperthyroid diagnosis. Furthermore, 19,391 individuals from the reference population were excluded as they had been registered with a cancer diagnosis before the index date. This left 95,469 hyperthyroid individuals (34,366 with GD, 3343 with GD and thyroid-associated orbitopathy, 24,713 with TNG, 33,040 with unspecified hyperthyroidism), and 364,494 reference individuals for analysis. The median follow-up time was 10.9 years (IQR 5.2-17.2) in the hyperthyroid population (HTP), and 11.2 years (5.4–17.4) in the reference population. Median days until receiving a cancer diagnosis was 2565 (984–4615) for hyperthyroid individuals and 2766 (1235–4739) for the reference individuals. Participant characteristics are summarized in Table 1.

**Table 1:** Characteristics of participants. Data are presented as median (IQR) or as *n* or as percent.

	Hyperthyroidism		Toxic nodular goiter		Graves’ disease	
	HTP	RP	HTP	RP	HTP	RP
*n*	95,462	364,578	32,854	124,447	43,396	167,308
Age (years) at HT diagnosis	57 (44–71)	58 (44–72)	63 (52–73)	62 (51–73)	55 (41–69)	54 (40–69)
Observation time (years)	10.9 (5.2–17.2)	11.2 (5.4–17.4)	11.4 (5.9–17.5)	11.5 (5.7–17.6)	12.6 (6.8–18.1)	12.9 (7–18.3)
Days until cancer diagnosis	2549 (984–4615)	2774 (1235–4739)	2642 (1108–4608)	2743 (1248–4,700)	2835 (1,192–4875)	2993 (1408–4941)
CCI, %						
0	80.6	86.7	81.1	85.4	83.6	88.6
1	12.5	9.0	12.6	9.8	11.1	7.8
2	3.7	2.4	3.5	2.8	2.9	2.1
>3	3.2	1.9	2.8	2.1	2.4	1.5

CCI, Charlson Comorbidity Index; HT, hyperthyroid; HTP, hyperthyroid population; IQR, interquartile range; *n*, total number of events; RP, reference population.

### Risk of cancer in hyperthyroid individuals

**Figure 1:**
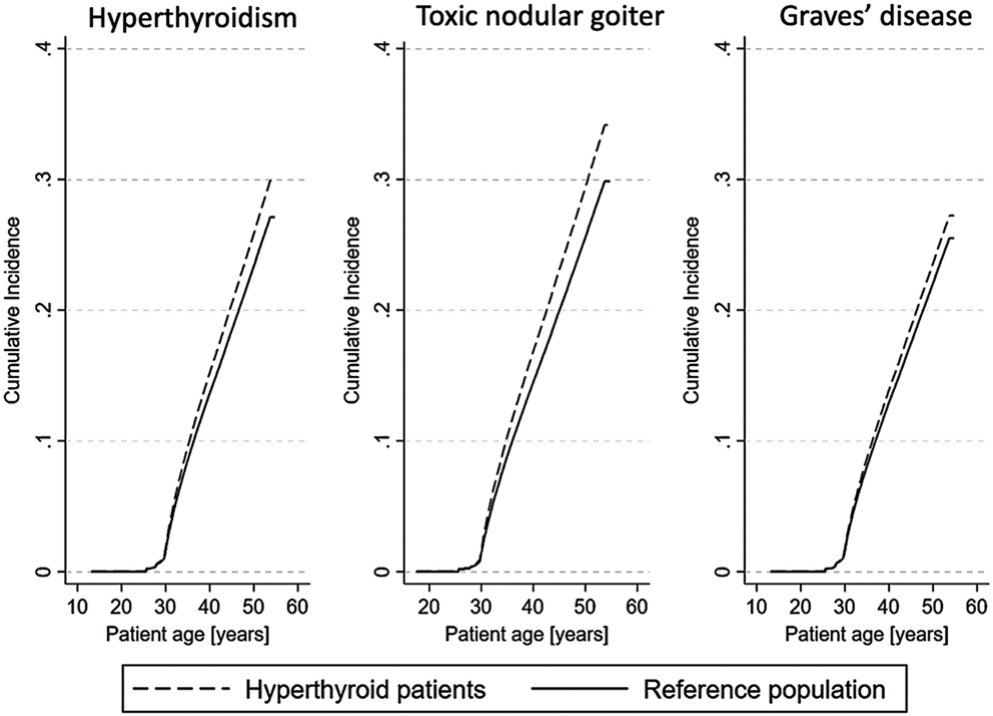
The cumulative incidence function based on the Fine and Gray competing risk regression models of cancer adjusted for Charlson Comorbidity Index in hyperthyroidism, TNG, and GD patients, compared with the reference population in the Danish National Patient Register. The cumulative incidence function is the likelihood of having the event (cancer), given that a competing event (death) has not yet been reached. The *y*-axis indicates the probability of cancer. The purpose of the log-log plot was to assess the assumption of proportionality. It does not reflect the sub-distribution hazard ratios which were calculated separately.

A total of 17,654 (18.5%) hyperthyroid individuals were diagnosed with cancer, compared to 59,977 (16.5%) individuals in the reference population. The frequency and risk of cancer are summarized in Table 2. Overall, irrespective of cause of hyperthyroidism and location of cancer, hyperthyroid patients had an increased risk of cancer (SHR: 1.13; 95% CI: 1.12–1.16). Almost similar risk estimates were achieved after adjusting for pre-existing morbidity (SHR: 1.12; 95% CI: 1.10–1.14). Stratification according to cause of hyperthyroidism showed that both GD and TNG were associated with an increased risk of all-cause cancer (Table 2) and that the risk was more pronounced in TNG than in GD. As outlined in Fig. 1, the cumulative incidence function, i.e. risk of having the event (cancer) at a certain time point – given that the participant has not met a competing event (death) – was higher in patients with hyperthyroidism, GD, and TNG, as compared with their respective reference populations.

**Table 2:** Risk of cancer in hyperthyroid individuals included in the cohort. Number of hyperthyroid patients and reference individuals registered with a cancer diagnosis. The crude competing risk regression model shows the sub-distribution hazard ratio (SHR) comparing the hyperthyroid population (HTP) to the reference population (RP), taking the competing risk of death (CRD) in hyperthyroid individuals into account. The adjusted for CCI shows the SHR comparing the hyperthyroid individuals to the reference population while adjusting for differences in Charlson Comorbidity Index (CCI).

	HTP, *n* (%)	RP, *n* (%)	CRD, SHR (95% CI)	Adj. CCI, SHR (95% CI)
Hyperthyroidism				
All cause cancer	17,654 (18.5)	59,977 (16.5)	1.13 (1.12–1.16)	1.12 (1.10–1.14)
Colorectal cancer	2384 (2.4)	8948 (2.2)	1.05 (1.01–1.10)	1.04 (0.99–1.09)
Lung cancer	2972 (3.0)	9458 (2.4)	1.24 (1.19–1.30)	1.20 (1.16–1.26)
Breast cancer	3546 (4.4)	12,939 (4.1)	1.07 (1.01–1.13)	1.07 (1.02–1.13)
Prostate cancer	954 (4.9)	3362 (4.4)	1.12 (1.04–1.20]	1.10 (1.02–1.19)
Toxic nodular goiter				
All cause cancer	7323 (22.3)	24,130 (19.4)	1.18 (1.15–1.21]	1.17 (1.14–1.20)
Colorectal cancer	1015 (2.9)	3751 (2.7)	1.07 (1.00–1.14)	1.06 (0.99-1.38)
Lung cancer	1294 (3.7)	3898 (2.8)	1.32 (1.24–1.41)	1.30 (1.22–1.38)
Breast cancer	1527 (5.4)	5269 (4.7)	1.40 (1.08–1.21)	1.14 (1.08–1.21)
Prostate cancer	370 (6.5)	1145 (5.1)	1.29 (1.14–1.45)	1.28 (1.14–1.44)
Graves’ disease				
All cause cancer	7785 (18.0)	27,617 (16.5)	1.09 (1.06–1.12)	1.08 (1.05–1.11)
Colorectal cancer	1029 (2.3)	4037 (2.3)	1.01 (0.94–1.01)	1.00 (0.93–1.07)
Lung cancer	1296 (2.9)	4304 (2.4)	1.19 (1.12–1.27)	1.17 (1.10–1.24)
Breast cancer	1655 (4.5)	6056 (4.2)	1.07 (1.01–1.13)	1.07 (1.02–1.35)
Prostate cancer	409 (5.1)	1474 (4.6)	1.10 (0.98–1.23)	1.08 (0.96–1.21)

Adj., adjusted; CRD, competing risk of death; HTP, hyperthyroid population; RP, reference population.

As summarized in Supplementary Table 2, after excluding individuals with non-melanoma skin cancer, a total of 15,962 (16.7%) hyperthyroid individuals and 53,477 (14.6%) reference individuals had a cancer diagnose. After adjusting for pre-existing morbidity, patients with hyperthyroidism (SHR: 1.16; 95% CI: 1.14–1.18), GD (SHR: 1.08; 95% CI: 1.05–1.11), and TNG (SHR: 1.19; 95% CI: 1.16–1.23) had an increased risk of cancer.

### Risk of cancer according to cancer localization

The frequency and risk of cancer according to localization is summarized in Table 2. Stratifying for cancer localization demonstrated an increased risk of lung, breast, and prostate cancer but not of colorectal cancer, in patients with hyperthyroidism as compared with the reference population. Further stratifying for cause of hyperthyroidism, the increased risk of prostate cancer was absent in male patients with GD but remained in patients with TNG.

### Influence of age at index date, sex, and censoring windows

The influence of age at index date, sex, and censoring windows is summarized in Supplementary Tables 3, 4, and 5, respectively. The analyses demonstrated a statistically significant increased risk of cancer in all age decades. Overall, the sex-stratified analyses yielded essentially similar results as the unstratified main analysis, i.e. increased risk of all cause cancer and lung cancer but not of colorectal cancer in either men or women. Compared to women, men had a significantly higher risk of all cause cancer and colorectal cancer, whereas the risk of lung cancer did not differ significantly between men and women. However, the increased risk of colorectal cancer in the male population was absent when taking the etiology of hyperthyroidism into account.

Applying a censoring window of 3 months, 1 year, or 3 years, respectively, did not significantly influence our findings.

## Discussion

By using Danish national health registries, we investigated the cancer risk in a nationwide patient cohort, thereby demonstrating a 12% increased relative risk of cancer in hyperthyroid individuals compared to the general population. Apart from a non-significant increased risk of prostate cancer in GD, our findings were robust in several supplementary analyses taking etiology of hyperthyroidism (GD or TNG), definition of primary (excluding non-melanoma skin cancer) and secondary outcome (cancer site – prostate, breast, lung, and colorectal), sex, and age at index date into consideration. Prostate cancer is more common in the elderly, and GD is less common than toxic nodular goiter in this age group. Therefore, the most likely explanation for the difference between GD and toxic nodular goiter and risk of prostate cancer is the lack of sufficient statistical power due to the relatively low number of patients with GD and prostate cancer.

Our finding of a higher cancer risk in males than in females is in line with existing knowledge (33). It has been demonstrated that males with hyperthyroidism more frequently experience treatment failure and are less likely to enter remission in case of GD (34). Since males are less frequent users of the health care system than females, one explanation for the higher cancer risk among males could be poorer adherence to treatment and lack of biochemical monitoring (35). We cannot qualify this statement as our databases do not contain thyroid function variables, choice and effect of treatment, and evaluation of compliance.

The mechanisms behind the association between hyperthyroidism and cancer are unknown. Our finding of a higher risk of cancer among patients with TNG compared to patients with GD is intriguing. Several theories can be proposed, but future studies must clarify whether the difference in cancer risk between TNG and GD is due to immunological factors, choice of treatment, epidemiological differences, or duration and severity of the hyperthyroid state.

Using a register-based approach on a large population-based sample, we have addressed some of the shortcomings in previous studies (13, 14, 16, 17, 18, 20, 21). When evaluating current evidence, it is important to realize that studies differ in their design (i.e. cross-sectional or prospective), study cohort (hospital or population based), diagnostic criteria (biochemical or register-based diagnosis), ascertainment of control population, handling of co-morbidity and confounders, length of follow-up, and age and gender distribution in the study population. Despite these diversities, many studies support that patients with current or previous hyperthyroidism are at increased risk of developing breast, lung, and prostate cancer, although exceptions exist (13).

It remains to be clarified how hyperthyroidism facilitates the development of cancer if such a link indeed exists. Numerous studies have shown thyroid hormones inducing *in vitro* proliferation of cancer cells of different types (3, 4, 5, 6, 7) as well as promoting *in vivo* tumor growth and angiogenesis (8, 9, 10, 11, 12), but the suggested mechanism varies. Integrin αvβ3, a plasma membrane protein predominantly expressed by rapidly proliferating cells, may play a key role (5, 8, 9). Thus, Meng *et al.* (5) demonstrated that thyroid hormones induced human lung cancer cell proliferation via crosstalk between integrin αvβ3 and estrogen receptor alpha. In a study in mice, thyroxine enhanced the weight of Lewis’ carcinoma and angiogenesis, but this required the activation of integrin αvβ3 (8). Regarding breast cancer, thyroid hormones seem to be involved in regulating the proliferation and gene expression of cancer cells through an interaction between the estradiol and triiodothyronine signaling systems (4). In fact, triiodothyronine may mimic or enhance the effects of estradiol on cancer proliferation (4). In another study in human breast cancer cells, thyroxine promoted cell proliferation through the estrogen receptor by a MAPK-dependent pathway (7). The observed increased cancer risk in hyperthyroid patients could be mediated or modified by the treatment modality (anti-thyroid drugs, radioiodine, and surgery). Very few studies, if any, have reported treatment-specific risk estimates for cancer in hyperthyroid patients, and current evidence is insufficient to draw any conclusion on this issue (13).

Hyperthyroidism is mostly caused by autoimmunity in GD or by one or more autonomously functioning thyroid nodules in TNG (36). Since these conditions differ with respect to age at onset, duration, and severity of the thyroid dysfunction (36), they may affect the risk of cancer differently. Hypothetically, an increased risk of cancer could pertain to autoimmunity per se rather than the hyperthyroid state or vice versa. However, the fact that our relative risk estimates for developing cancer was increased in both TNG and GD support the assumption that cancer risk is not attributed to autoimmune factors alone and could at least partly be driven by the hyperthyroid state.

Our study has several strengths. First, by using the DNPR cohort, we included all adult patients having or having had hyperthyroidism with contact to the health care system in Denmark. This provided a large cohort of participants with high capture and nearly complete follow-up. Second, the impact of Berkson’s bias was minimized as a confounding factor by applying several censoring windows. By this approach, our findings were not significantly changed, thus making reverse causality unlikely. Third, we used the Fine & Gray semiparametric risk regression model in the risk evaluation, thereby taking the competing risk of death in hyperthyroid patients into account (30). Thus, we have addressed several shortcomings seen in previous studies.

Some limitations should be mentioned. First, although we adjusted for a number of risk factors, covariates, such as alcohol intake and smoking habits, were unavailable to us. Second, register-based studies are dependent on the validity of the data provided by the clinicians in their everyday clinical practice. However, several studies have shown that the positive predictive value of diagnoses registered in the DNPR is high (36). Nevertheless, individuals in the general population may have undiagnosed hyperthyroidism. If the fraction of such individuals is significant, the SHRs in our study are underestimated, and therefore does not affect our conclusion. Third, as information regarding treatment (i.e. antithyroid drug, radioiodine, and surgery), disease severity in terms of thyroid function tests, and duration of hyperthyroidism is lacking, we could not explore how the severity and duration of hyperthyroidism and its management affected the risk of cancer.

In conclusion, this nationwide register-based data demonstrated a 12% increased risk of cancer in a large cohort of hyperthyroid patients. The risk depended on the type of cancer, with increased SHRs for prostate, breast, and lung cancer but not for colorectal cancer. Similar results were found when stratifying for cause of hyperthyroidism. Although the validity of our results is supported by the size of the study it remains to be proven that hyperthyroidism is a pro-cancerogenic condition.

## Supplementary Materials

Table S1. Exposure variables and outcome variables

Table S2. Risk of cancer in hyperthyroid individuals included in the cohort, excluding non-melanoma skin cancer.

Table S3. Risk of all-cause cancer in hyperthyroid individuals, according to age at index

Table S4. Risk of cancer in hyperthyroid individuals included in the cohort, gender-stratified.

Table S5. Risk of all-cause cancer in hyperthyroid individuals, using censoring windows.

## Declaration of interest

The authors declare that there is no conflict of interest that could be perceived as prejudicing the impartiality of the study reported. Steen J Bonnema is an editorial board member of *European Thyroid Journal*. Steen J Bonnema was not involved in the review or editorial process for this paper, on which he is listed as an author.

## Funding

Thea Riis was supported by a research grant from ‘Fonden af 17-12-1981http://dx.doi.org/10.13039/501100008247’. Steen J Bonnema was supported by a grant from Desirée and Niels Yde Foundation.

## Author contribution statement

TR is the guarantor, and as the article’s lead author she had full access to all the data in the study, takes responsibility for the work and conduct of the study, and controlled the decision to publish. TR affirms that the manuscript is an honest, accurate, and transparent account of the study being reported; that no important aspects of the study have been omitted; and that any discrepancies from the study as planned (and, if relevant, registered) have been explained. TR drafted the initial manuscript and SJB, THB, and LF critically revised the manuscript for important intellectual content. Final approval of the version to be published was given by all authors. The corresponding author attests that all listed authors meet the authorship criteria and that no others meeting the criteria have been omitted.

## References

[1] GBD 2017 Causes of Death Collaborators. Global, regional, and national age-sex-specific mortality for 282 causes of death in 195 countries and territories, 1980–2017: a systematic analysis for the Global Burden of Disease Study 2017. Lancet 2018 392 1736–1788. (10.1016/S0140-6736(1832203-7) 30496103 PMC6227606

[2] Engholm G Ferlay J Christensen N Bray F Gjerstorff ML Klint A Køtlum JE Olafsdóttir E Pukkala E & Storm HH. NORDCAN–a Nordic tool for cancer information, planning, quality control and research. Acta Oncologica 2010 49 725–736. (10.3109/02841861003782017) 20491528

[3] Dinda S Sanchez A & Moudgil V. Estrogen-like effects of thyroid hormone on the regulation of tumor suppressor proteins, p53 and retinoblastoma, in breast cancer cells. Oncogene 2002 21 761–768. (10.1038/sj.onc.1205136) 11850804

[4] Hall LC Salazar EP Kane SR & Liu N. Effects of thyroid hormones on human breast cancer cell proliferation. Journal of Steroid Biochemistry and Molecular Biology 2008 109 57–66. (10.1016/j.jsbmb.2007.12.008) 18328691

[5] Meng R Tang HY Westfall J London D Cao JH Mousa SA Luidens M Hercbergs A Davis FB Davis PJ, *et al.* Crosstalk between integrin αvβ3 and estrogen receptor-α is involved in thyroid hormone-induced proliferation in human lung carcinoma cells. PLoS One 2011 6 e27547. (10.1371/journal.pone.0027547) 22132110 PMC3222665

[6] Mousa SA Yalcin M Bharali DJ Meng R Tang HY Lin HY Davis FB & Davis PJ. Tetraiodothyroacetic acid and its nanoformulation inhibit thyroid hormone stimulation of non-small cell lung cancer cells in vitro and its growth in xenografts. Lung Cancer 2012 76 39–45. (10.1016/j.lungcan.2011.10.003) 22024450

[7] Tang HY Lin HY Zhang S Davis FB & Davis PJ. Thyroid hormone causes mitogen-activated protein kinase-dependent phosphorylation of the nuclear estrogen receptor. Endocrinology 2004 145 3265–3272. (10.1210/en.2004-0308) 15059947

[8] Carmona-Cortés J Rodríguez-Gómez I Wangensteen R Banegas I García-Lora ÁM Quesada A Osuna A & Vargas F. Effect of thyroid hormone-nitric oxide interaction on tumor growth, angiogenesis, and aminopeptidase activity in mice. Tumour Biology 2014 35 5519–5526. (10.1007/s13277-014-1726-2) 24549786

[9] Cayrol F Díaz Flaqué MC Fernando T Yang SN Sterle HA Bolontrade M Amorós M Isse B Farías RN Ahn H, *et al.* Integrin αvβ3 acting as membrane receptor for thyroid hormones mediates angiogenesis in malignant T cells. Blood 2015 125 841–851. (10.1182/blood-2014-07-587337) 25488971 PMC4311229

[10] Mousa SA Davis FB Mohamed S Davis PJ & Feng X. Pro-angiogenesis action of thyroid hormone and analogs in a three-dimensional in vitro microvascular endothelial sprouting model. International Angiology 2006 25 407–413. 17164749

[11] Sterle HA Valli E Cayrol F Paulazo MA Martinel Lamas DJ Diaz Flaqué MC Klecha AJ Colombo L Medina VA Cremaschi GA, *et al.* Thyroid status modulates T lymphoma growth via cell cycle regulatory proteins and angiogenesis. Journal of Endocrinology 2014 222 243–255. (10.1530/JOE-14-0159) 24928937

[12] Vermey ML Marks GT & Baldridge MG. Effect of thyroid function on MNU-induced mammary carcinogenesis. Zoological Science 2015 32 272–277. (10.2108/zs140124) 26003983

[13] Tran TV Kitahara CM de Vathaire F Boutron-Ruault MC & Journy N. Thyroid dysfunction and cancer incidence: a systematic review and meta-analysis. Endocrine-Related Cancer 2020 27 245–259. (10.1530/ERC-19-0417) 32045361

[14] Chan YX Knuiman MW Divitini ML Brown SJ Walsh J & Yeap BB. Lower TSH and higher free thyroxine predict incidence of prostate but not breast, colorectal or lung cancer. European Journal of Endocrinology 2017 177 297–308. (10.1530/EJE-17-0197) 28684452

[15] Hellevik AI Asvold BO Bjøro T Romundstad PR Nilsen TI & Vatten LJ. Thyroid function and cancer risk: a prospective population study. Cancer Epidemiology, Biomarkers and Prevention 2009 18 570–574. (10.1158/1055-9965.EPI-08-0911) 19155436

[16] Khan SR Chaker L Ruiter R Aerts JG Hofman A Dehghan A Franco OH Stricker BH & Peeters RP. Thyroid function and cancer risk: the Rotterdam study. Journal of Clinical Endocrinology and Metabolism 2016 101 5030–5036. (10.1210/jc.2016-2104) 27648963

[17] Krashin E Silverman B Steinberg DM Yekutieli D Giveon S Fabian O Hercbergs A Davis PJ Ellis M & Ashur-Fabian O. Opposing effects of thyroid hormones on cancer risk: a population-based study. European Journal of Endocrinology 2021 184 477–486. (10.1530/EJE-20-1123) 33444229

[18] Søgaard M Farkas DK Ehrenstein V Jørgensen JO Dekkers OM & Sørensen HT. Hypothyroidism and hyperthyroidism and breast cancer risk: a nationwide cohort study. European Journal of Endocrinology 2016 174 409–414. (10.1530/EJE-15-0989) 26863886

[19] Hassan MM Kaseb A Li D Patt YZ Vauthey JN Thomas MB Curley SA Spitz MR Sherman SI Abdalla EK, *et al.* Association between hypothyroidism and hepatocellular carcinoma: a case-control study in the United States. Hepatology 2009 49 1563–1570. (10.1002/hep.22793) 19399911 PMC3715879

[20] Kirkegård J Farkas DK Jørgensen JOL & Cronin-Fenton D. Hyper- and hypothyroidism and gastrointestinal cancer risk: a Danish nationwide cohort study. Endocrine Connections 2018 7 1129–1135. (10.1530/EC-18-0258) 30352404 PMC6215792

[21] Mellemgaard A From G Jørgensen T Johansen C Olsen JH & Perrild H. Cancer risk in individuals with benign thyroid disorders. Thyroid 1998 8 751–754. (10.1089/thy.1998.8.751) 9777744

[22] Pedersen CB. The Danish civil registration system. Scandinavian Journal of Public Health 2011 39(Supplement) 22–25. (10.1177/1403494810387965) 21775345

[23] Thygesen LC Daasnes C Thaulow I & Brønnum-Hansen H. Introduction to Danish (nationwide) registers on health and social issues: structure, access, legislation, and archiving. Scandinavian Journal of Public Health 2011 39(Supplement) 12–16. (10.1177/1403494811399956) 21898916

[24] Schmidt M Schmidt SA Sandegaard JL Ehrenstein V Pedersen L & Sørensen HT. The Danish National Patient Registry: a review of content, data quality, and research potential. Clinical Epidemiology 2015 7 449–490. (10.2147/CLEP.S91125) 26604824 PMC4655913

[25] Kitahara CM Kӧrmendiné Farkas D Jørgensen JOL Cronin-Fenton D & Sørensen HT. Benign thyroid diseases and risk of thyroid cancer: a nationwide cohort study. Journal of Clinical Endocrinology and Metabolism 2018 103 2216–2224. (10.1210/jc.2017-02599) 29590402 PMC6276704

[26] Yeh NC Chou CW Weng SF Yang CY Yen FC Lee SY Wang JJ & Tien KJ. Hyperthyroidism and thyroid cancer risk: a population-based cohort study. Experimental and Clinical Endocrinology and Diabetes 2013 121 402–406. (10.1055/s-0033-1341474) 23616188

[27] Christensen S Johansen MB Christiansen CF Jensen R & Lemeshow S. Comparison of Charlson comorbidity index with SAPS and Apache scores for prediction of mortality following intensive care. Clinical Epidemiology 2011 3 203–211. (10.2147/CLEP.S20247) 21750629 PMC3130905

[28] Austin PC & Fine JP. Practical recommendations for reporting Fine-Gray model analyses for competing risk data. Statistics in Medicine 2017 36 4391–4400. (10.1002/sim.7501) 28913837 PMC5698744

[29] Brandt F Thvilum M Almind D Christensen K Green A Hegedüs L & Brix TH. Morbidity before and after the diagnosis of hyperthyroidism: a nationwide register-based study. PLoS One 2013 8 e66711. (10.1371/journal.pone.0066711) 23818961 PMC3688572

[30] Schuster NA Hoogendijk EO Kok AAL Twisk JWR & Heymans MW. Ignoring competing events in the analysis of survival data may lead to biased results: a nonmathematical illustration of competing risk analysis. Journal of Clinical Epidemiology 2020 122 42–48. (10.1016/j.jclinepi.2020.03.004) 32165133

[31] de Menezes RF Bergmann A & Thuler LC. Alcohol consumption and risk of cancer: a systematic literature review. Asian Pacific Journal of Cancer Prevention 2013 14 4965–4972. (10.7314/apjcp.2013.14.9.4965) 24175760

[32] O'Keeffe LM Taylor G Huxley RR Mitchell P Woodward M & Peters SAE. Smoking as a risk factor for lung cancer in women and men: a systematic review and meta-analysis. BMJ Open 2018 8 e021611. (10.1136/bmjopen-2018-021611) PMC6194454 30287668

[33] Jackson SS Marks MA Katki HA Cook MB Hyun N Freedman ND Kahle LL Castle PE Graubard BI & Chaturvedi AK. Sex disparities in the incidence of 21 cancer types: quantification of the contribution of risk factors. Cancer 2022 128 3531–3540. (10.1002/cncr.34390) 35934938 PMC11578066

[34] Allahabadia A Daykin J Holder RL Sheppard MC Gough SC & Franklyn JA. Age and gender predict the outcome of treatment for Graves' hyperthyroidism. Journal of Clinical Endocrinology and Metabolism 2000 85 1038–1042. (10.1210/jcem.85.3.6430) 10720036

[35] Janssen EM Dy SM Meara AS Kneuertz PJ Presley CJ & Bridges JFP. Analysis of patient preferences in lung cancer - estimating acceptable tradeoffs between treatment benefit and side effects. Patient Prefer Adherence 2020 14 927–937. (10.2147/PPA.S235430) 32581519 PMC7276327

[36] Thygesen SK Christiansen CF Christensen S Lash TL & Sørensen HT. The predictive value of ICD-10 diagnostic coding used to assess Charlson comorbidity index conditions in the population-based Danish National Registry of Patients. BMC Medical Research Methodology 2011 11 83. (10.1186/1471-2288-11-83) 21619668 PMC3125388

