# Supplementary material for: Hyperthyroidism and the risk of non-thyroid cancer: a Danish register-based long-term follow-up study
Source: Eur Thyroid J. 2024 Apr 1;13(2):e230181. doi: 10.1530/ETJ-23-0181 (PMC11046354; doi:10.1530/ETJ-23-0181)
Supplement: Table S1. Exposure variables and outcome variables [file supplementary_table_1.pdf]

Table S1. Exposure variables and outcome variables

Disease/condition	*Data source*	*Codes*	*Variables*
Exposure groups			
Hyperthyroidism	DNPR	ICD-10	E05.0, E05.1, E05.2, E05.9 E05.3, E05.4, E05.5, E05.8 + E06.2, E06.4 + H05.2 (excluding H052A)
Graves' disease	DNPR	ICD-10	E05.0
Toxic nodular goiter	DNPR	ICD-10	E05.1, E05.2
Primary endpoint			
All-cause cancer	DNPR	ICD-10	C00, C01, C02, C03, C04, C05, C06, C07, C08, C09, C10, C11, C12, C13, C14, C30, C31, C15, C16, C17, C18, C19, C20, C21, C22, C23, C25, C32, C33, C34, C38 (exclude 380), C37, C40, C43, C44, C45, C46, C47, C48, C49, C50, C51, C52, C53, C54, C55, C56, C58, C60, C61, C62, C64, C65, C66, C67, C69, C70, C71, C72, C74, C75, C26, C39, C41, C57, C63, C68, C76, C80, C85, C96, C81, C82, C83, C84, C88, C90, C92, C93, C94, C95, D45, D46, C97, D37, D38, D39, D40, D41, D42, D43, D44, D47, D48, (exclude D440), C77, C780, C781, C782, C783, C784, C785, C787, C788, C790, C791, C792, C793, C794, C795, C796, C797, C798, C80
Secondary endpoints			
Colorectal cancer	DNPR	ICD-10	C18, C20
Lung cancer	DNPR	ICD-10	C34
Breast cancer	DNPR	ICD-10	C50
Prostate cancer	DNPR	ICD-10	C61
CCI	DNPR	ICD-10	410, I21, I22, I23, 42709, 42710, 42711, 42719, 42899, 78249, I50, I110, I130, I132, 440, 441, 442, 443, 444, 445, I70, I72, I73, I74, I77, 43, I6, G45, G46 (exclude 439), 29009, 2901, 29309, F00, F01, F02, F03, F051, G30, 490, 491, 493, 515, 516, 517, 518, J40, J41, J42, J43, J44, J45, J46, J47, J60, J61, J62, J63, J64, J65, J66, J67, J684, J701, J703, J841, J920, J961, J982, J983, 712, 716, 734, 446, 13599, M05, M06, M08, M09, M30, M31, M32, M33, M34, M35, M36, D86, 53091, 53098, 531, 532, 533, 534, K221, K25, K26, K27, K28, 571, 57301, 57304, B18, K700, K701, K702, K703, K709, K71, K73, K74, K760, 24900, 24906, 24907, 24909, 25000, 25006, 25007, 25009, E100, E101, E110, E111, E119, 344, G81, G82, 403, 404, 580, 582, 583, 584, 59009, 59319, 7531, 792, I12, I13, N00, N01, N02, N03, N04, N05, N07, N11, N14, N17, N18, N19, Q61, 24901, 24902, 24903, 24904, 24905, 24908, 25001, 25002, 25003, 25004, 25005, 25008, E102, E103, E104, E105, E107, E108, E112, E113, E114, E115, E116, E117, E118, 07000, 07002, 07004, 07006, 07008, 57300, 4560, B150, B160, B162, B190, K704, K72, K766, I85, B21, B22, B23, B24

The table shows the ICD-10 codes used to define diseases or conditions that were the focus of the study. CCI, Charlson Comorbidity Index; DNPR, Danish National Patient Register; ICD-10, World Health Organization International Classification of Diseases version 10.
