# Supplementary material for: Hyperthyroidism and the risk of non-thyroid cancer: a Danish register-based long-term follow-up study
Source: Eur Thyroid J. 2024 Apr 1;13(2):e230181. doi: 10.1530/ETJ-23-0181 (PMC11046354; doi:10.1530/ETJ-23-0181)
Supplement: Table S2. Risk of cancer in hyperthyroid individuals included in the cohort, excluding non-melanoma skin cancer. [file supplementary_table_2.pdf]

Table S2. Risk of cancer in hyperthyroid individuals included in the cohort, excluding non-melanoma skin cancer.

	Hyperthyroid population N (%)	Reference population N (%)	Competing risk of death (SHR [95% CI])	Adjusted for CCI (SHR [95% CI])
**Hyperthyroidism**				
All cause cancer	15,962 (16.7)	53,477 (14.6)	1.16 [1.14-1.18]	1.16 [1.14-1.18]
**Toxic nodular goiter**				
All cause cancer	6,319 (20.5)	20,458 (17.4)	1.20 [1.17-1.23]	1.19 [1.16-1.23]
**Graves' disease**				
All cause cancer	6,486 (16.1)	22,961 (14.7)	1.10 [1.07-1.13]	1.08 [1.05-1.11]

Number of hyperthyroid patients and reference individuals registered with a cancer diagnosis. The crude competing risk regression model shows the sub-distribution hazard ratio (SHR) comparing the hyperthyroid individuals to the reference population, taking the competing risk of death in hyperthyroid individuals into account. The adjusted for CCI shows the SHR comparing the hyperthyroid individuals to the reference population while adjusting for differences in Charlson Comorbidity Index (CCI).
