# Supplementary material for: Hyperthyroidism and the risk of non-thyroid cancer: a Danish register-based long-term follow-up study
Source: Eur Thyroid J. 2024 Apr 1;13(2):e230181. doi: 10.1530/ETJ-23-0181 (PMC11046354; doi:10.1530/ETJ-23-0181)
Supplement: Table S3. Risk of all-cause cancer in hyperthyroid individuals, according to age at index [file supplementary_table_3.pdf]

Table S3. Risk of all-cause cancer in hyperthyroid individuals, according to age at index

	Hyperthyroid population N (%)	Reference population N (%)	Competing risk of death (SHR [95% CI])	Adjusted for CCI (SHR [95% CI])
age <50	2,630 (8.3)	9,190 (7.1)	1.14 [1.09-1.19]	1.13 [1.09-1.18]
age 50-59	3,909 (21.7)	13,038 (18.7)	1.18 [1.14-1.22]	1.18 [1.14-1.22]
age 60-69	4,878 (27.0)	16,979 (24.9)	1.10 [1.07-1.14]	1.10 [1.06-1.14]
age 70-79	4,440 (25.5)	15,253 (25.5)	1.14 [1.12-1.16]	1.08 [1.05-1.12]
age 80-	1,791 (17.5)	5,480 (14.8)	1.22 [1.15-1.29]	1.20 [1.13-1.27]

Number of hyperthyroid patients and reference individuals registered with a cancer diagnosis, n (%); total number of events and percent of the total population. The study population is divided into age groups according to age at index. The crude competing risk regression model shows the SHR comparing the hyperthyroid individuals to the reference population in the corresponding age group, taking the competing risk of death in hyperthyroid individuals into account. The adjusted for CCI shows the SHR comparing the hyperthyroid individuals to the reference population in the corresponding age group while adjusting for differences in CCI.
