# Supplementary material for: Hyperthyroidism and the risk of non-thyroid cancer: a Danish register-based long-term follow-up study
Source: Eur Thyroid J. 2024 Apr 1;13(2):e230181. doi: 10.1530/ETJ-23-0181 (PMC11046354; doi:10.1530/ETJ-23-0181)
Supplement: Table S4. Risk of cancer in hyperthyroid individuals included in the cohort, gender-stratified. [file supplementary_table_4.pdf]

Table S4. Risk of cancer in hyperthyroid individuals included in the cohort, gender-stratified.

	Hyperthyroid population N (%)	Reference population N (%)	Competing risk of death (SHR [95% CI])	Adjusted for CCI (SHR [95% CI])
**Hyperthyroidism**				
All cause cancer				
Men	3,920 (21.3)	12,363 (17.6)	1.25 [1.20-1.29]	1.19 [1.15-1.24]
Women	13,741 (17.8)	47,511 (16.2)	1.12 [1.09-1.14]	1.12 [1.10-1.14]
Colorectal cancer				
Men	557 (2.8)	1,926 (2.5)	1.24 [0.96-1.32]	1.11 [1.01-1.22]
Women	1,815 (2.2)	7,013 (2.2)	1.02 [0.97-1.08]	1.01 [0.96-1.07]
Lung cancer				
Men	693 (3.5)	2,047 (2.6)	1.34 [1.22-1.46]	1.24 [1.14-1.35]
Women	2,281 (2.8)	7,414 (2.3)	1.22 [1.16-1.28]	1.19 [1.14-1.25]
Breast cancer				
Women	3,546 (4.4)	12,939 (4.1)	1.07 [1.01-1.13]	1.07 [1.02-1.13]
Prostate cancer				
Men	954 (4.9)	3,362 (4.4)	1.12 [1.04-1.20]	1.10 [1.02-1.19]
**Toxic nodular goiter**				
All cause cancer				
Men	1,331 (26.2)	4,081 (21.2)	1.25 [1.20-1.29]	1.26 [1.18-1.34]
Women	5,645 (22.1)	18,690 (19.3)	1.17 [1.13-1.20]	1.16 [1.13-1.20]
Colorectal cancer				
Men	192 (3.3)	673 (3.0)	1.29 [1.20-1.37]	1.10 [0.94-1.29]
Women	817 (2.8)	3,079 (2.7)	1.05 [0.97-1.13]	1.05 [0.97-1.13]
Lung cancer				
Men	239 (4.1)	698 (3.0)	1.36 [1.17-1.57]	1.30 [1.12-1.51]
Women	1,057 (3.6)	3,211 (2.8)	1.31 [1.22-1.41]	1.29 [1.21-1.39]
Breast cancer				
Women	1,527 (5.4)	5,269 (4.7)	1.40 [1.08-1.21]	1.14 [1.08-1.21]
Prostate cancer				
Men	370 (6.5)	1,145 (5.1)	1.29 [1.14-1.45]	1.28 [1.14-1.44]
**Graves' disease**				
All cause cancer				
Men	5,508 (18.5)	1,641 (21.2)	1.17 [1.10-1.24]	1.14 [1.07-1.20]
Women	6,150 (17.3)	22,137 (16.1)	1.07 [1.04-1.10]	1.07 [1.04-1.10]
Colorectal cancer				
Men	227 (2.8)	832 (2.6)	1.08 [0.93-1.25]	1.05 [0.90-1.22]
Women	788 (2.13)	3,202 (2.2)	0.97 [0.90-1.05]	0.96 [0.89-1.04]
Lung cancer				

Men	305 (3.8)	815 (2.9)	1.32 [1.16-1.50]	1.26 [1.10-1.43]
Women	996 (2.7)	3,389 (2.3)	1.17 [1.09-1.25]	1.15 [1.07-1.23]
Breast cancer				
Women	1,655 (4.5)	6,056 (4.2)	1.07 [1.01-1.13]	1.07 [1.02-1.35]
Prostate cancer				
Men	409 (5.1)	1,474 (4.6)	1.10 [0.98-1.23]	1.08 [0.96-1.21]

Number of hyperthyroid patients and reference individuals registered with a cancer diagnosis. The crude competing risk regression model shows the sub-distribution hazard ratio (SHR) comparing the hyperthyroid individuals to the reference population, taking the competing risk of death in hyperthyroid individuals into account. The adjusted for CCI shows the SHR comparing the hyperthyroid individuals to the reference population while adjusting for differences in Charlson Comorbidity Index (CCI).
