# Supplementary material for: Hyperthyroidism and the risk of non-thyroid cancer: a Danish register-based long-term follow-up study
Source: Eur Thyroid J. 2024 Apr 1;13(2):e230181. doi: 10.1530/ETJ-23-0181 (PMC11046354; doi:10.1530/ETJ-23-0181)
Supplement: Table S5. Risk of all-cause cancer in hyperthyroid individuals, using censoring windows. [file supplementary_table_5.pdf]

Table S5. Risk of all-cause cancer in hyperthyroid individuals, using censoring windows.

	Hyperthyroid population N (%)	Reference population N (%)	Competing risk of death (SHR [95% CI])	Adjusted for CCI (SHR [95% CI])
3 months censoring	16,688 (17.7)	58,025 (16.1)	1.11 [1.10-1.13]	1.10 [1.08-1.12]
1 year censoring	15,370 (16.5)	53,684 (15.3)	1.10 [1.08-1.12]	1.09 [1.07-1.11]
3 years censoring	12,886 (14.2)	43,934 (13.1)	1.10 [1.07-1.12]	1.10 [1.08-1.12]

Number of hyperthyroid patients and reference individuals registered with a cancer diagnosis, n (%); total number of events and percent of the total population, adjusting for Berkson's bias. The crude competing risk regression model shows the sub-distribution hazard ratio (SHR) comparing the hyperthyroid individuals to the reference population, taking the competing risk of death in hyperthyroid individuals into account. The adjusted for CCI shows the SHR comparing the hyperthyroid individuals to the reference population group while adjusting for differences in CCI.
